# A Fractional-Order Partially Non-Linear Model of a Laboratory Prototype of Hydraulic Canal System

**DOI:** 10.3390/e21030309

**Published:** 2019-03-21

**Authors:** Saddam Gharab, Vicente Feliu-Batlle, Raul Rivas-Perez

**Affiliations:** 1 Instituto de Investigaciones Energéticas y Aplicaciones Industriales, Universidad de Castilla-La Mancha, 13071 Ciudad Real, Spain; 2 Escuela Técnica Superior de Ingenieros Industriales, Universidad de Castilla-La Mancha, 13071 Ciudad Real, Spain; 3 Departamento de Automática y Computación Universidad Tecnológica de la Habana, CUJAE, La Habana 19390, Cuba

**Keywords:** PRBS, fractional-order dynamic models, hydraulic canal system, nonlinear models, time-domain identification

## Abstract

This article addresses the identification of the nonlinear dynamics of the main pool of a laboratory hydraulic canal installed in the University of Castilla La Mancha. A new dynamic model has been developed by taking into account the measurement errors caused by the different parts of our experimental setup: (a) the nonlinearity associated to the input signal, which is caused by the movements of the upstream gate, is avoided by using a nonlinear equivalent upstream gate model, (b) the nonlinearity associated to the output signal, caused by the sensor’s resolution, is avoided by using a quantization model in the identification process, and (c) the nonlinear behaviour of the canal, which is related to the working flow regime, is taken into account considering two completely different models in function of the operating regime: the free and the submerged flows. The proposed technique of identification is based on the time-domain data. An input pseudo-random binary signal (PRBS) is designed depending on the parameters of an initially estimated linear model that was obtained by using a fundamental technique of identification. Fractional and integer order plus time delay models are used to approximate the responses of the main pool of the canal in its different flow regimes. An accurate model has been obtained, which is composed of two submodels: a first order plus time delay submodel that accurately describes the dynamics of the free flow and a fractional-order plus time delay submodel that properly describes the dynamics of the submerged flow.

## 1. Introduction

Improving the management of the scarce available water resources is a most important research area because of the strong dependence of the mankind on fresh water [1]. Some updated statistics show that irrigated agriculture represents the largest consumer of fresh water that consumes a total percentage of 70% of all the available fresh water [2,3]. In those systems, water is transported and distributed through long distances by using irrigation main canals, which have huge water losses. Several researches have focused on improving the management and efficiency of hydraulic canals by means of introducing electronics and automation in these civil infraestructures. This objective has therefore a high scientific, economic and social interest [4]. Although the establishment of dynamic models of the process to be controlled is of utmost importance in the design of automation systems, modelization of the dynamics of main irrigation canals has a lower level of development than modelization of other hydraulic infraestructures like dams, piping, hydroelectric plants and drinking water supplies to towns [5]. In particular, the obtention of simple dynamic models that accurately reproduce the nonlinear and distributed nature of main irrigation canals is of great interest in order to design high performance controllers that allow an efficient automation of these systems.

Models of hydraulic canals can be obtained from the well-known Saint-Venant equations [6,7,8] or from the use of system identification tools [9,10,11]. The most important approaches to the identification of main canal pools have been reviewed in [12]. These include a linearization of the hydraulic canal model based on the highly nonlinear Saint-Venant differential equations, an equivalent linear infinite order transfer function, a second order equivalent non-linear transfer function, a finite order linear state-space model, a finite order linear transfer function, a neural network model, a fuzzy model and a petri net model, etc., e.g., [13]. Among the most popular linear models of canals, we mention the integrator model with delay (ID) for a pool of a main irrigation canal under backwater flow conditions proposed by Schuurmans. This same model has also been used to develop state-space MIMO models of complete canals in [14,15]. Other improvements have been proposed to the ID model by Litrico in [16]. Nonlinear irrigation canal models have also been developed in [17,18].

Applying the system identification tools, a non-linear model based on RNA with NARX structure was obtained from the distribution of water in a section of a main irrigation canal. The validation results of such model show that it reproduces with a high degree of adequacy the dynamic nonlinear behavior of the considered system, even considering data in real time that not used in the training of RNA [13]. However, this work did not consider changes in the operating flow regime.

Recently, fractional calculus has become a powerful tool to model and control real industrial processes [19,20,21]. In particular, it has showed interesting advantages when modelling dynamic systems of distributed nature, i.e., described by partial differential equations, as the already mentioned Saint-Venant equations are. By using this mathematical tool, the distributed dynamics of these systems, which are often represented by high order integer models, can be concentrated in simple fractional-order models with an equivalent accuracy. This tool has been successfully applied to model electrochemical [22], thermal [23] or hydraulic processes [24].

Several research works have been carried out by our group on the characterization of fractional-order dynamics in main irrigation canals. We have used the hydraulic canal system installed in the laboratory of the Mechanics of Fluids of the University of Castilla La Mancha. In a first work, a fractional-order model with a time delay was characterized for the main pool of the canal in [25], by means of a direct system identification approach [26] that allows the immediate derivation of a continuous-time model using continuous-time model identification tools. Later, the two pools that constitute the canal were identified and a TITO (two input/two output) fractional-order model was obtained in [27] that took into account the coupling existing between the two pools. In [28] the combination of the TITO model with a water level closed-loop control of the upstream pool of our canal allowed to reproduce the experimental results with a higher fidelity (up to a 50% of improvement) than the linearized integer-order MIMO models traditionally proposed in the scientific literature. Based on these fractional-order models, a fractional order Wiener-Hopf optimal control system was designed and tested in [29], that efficiently adjusted the downstream end water level of the main canal pool.

However, the previous works yielded fractional-order linear models that reproduced the process dynamics around an operating flow regime. Since canals are inherently nonlinear systems, a change in the operating regime would yield a completely different dynamic model. Then these models are not valid to reproduce the canal dynamics when large changes are produced in the operating setpoint, and more elaborate nonlinear models are required.

Moreover, the dynamics of a canal may sharply change depending on the geometry of the structure and the flow characteristics [6]. Such hydraulic structures may present two different regimes. The first one is characterized by the free flow condition and the flow is denoted critical or supercritical. In this case, the downstream water level has no influence on the flow and the discharge only depends on the upstream water level. The second regime is characterized by the submerged flow condition and the flow is denoted subcritical. In this case, the flow could be influenced by the downstream water level. Accordingly, the responses of the system under each kind of flow have to be modelled by different nonlinear dynamic models.

This paper proposes therefore a new nonlinear model to describe the dynamics of the principal pool of a main irrigation canal. A model identification procedure based on time-domain data is proposed. The identification works previously carried out in the hydraulic canal system have shown that the step responses presents high-frequency vibrations that could not be identified using the most elementary techniques of identification (responses to impulses, steps or ramps). Then a pseudo-random binary signal is designed in function of the system parameters and the prototype characteristics in order to identify the behaviour of the system in a broad range of frequency. The normalized root mean square error between the process output and the fitted model response is used as performance index to be minimized in the identification process. This index has been extensively used to describe the performance of the system approximations, being it independent of scale factors or the number of data [30].

This paper is organized as follows. Section 2 gives a brief description of the laboratory hydraulic canal prototype. Section 3 presents the proposed dynamic models. Section 4 illustrates the progress achieved in the data processing system of the hydraulic canal in order to improve the precision of the different measured signals and develops the new identification technique used to obtain the parameters of the nonlinear models. Section 5 presents the experimental data used when identifying the dynamic of our system and define the fitting indexes. Section 6 and Section 7 present the different identified models and stress the improvements obtained by using the new technique of identification jointly with the new nonlinear models proposed for our laboratory setup. Finally Section 8 gives some conclusions.

## 2. Laboratory Hydraulic Canal System

**Figure 1:**
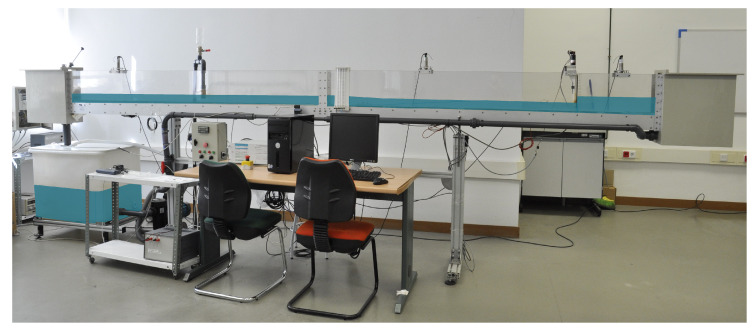
Hydraulic canal prototype of the university of Castilla La Mancha.

The system considered in this paper is a platform of an hydraulic canal system installed in the School of Industrial Engineering of the University of Castilla-La Mancha, located in Ciudad Real. As it is detailed in Figure 1, such a system is characterized by its variable slope rectangular canal with glass walls. Their dimensions are like, 5 m long, 0.08 m wide and 0.25 m walls high.

**Figure 2:**
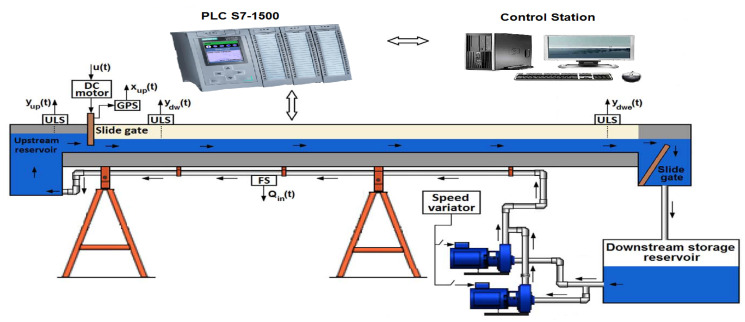
Schematic representation of the prototype hydraulic canal.

In order to avoid any waste of water, the water flows inside the canal in a closed loop circuit. The schematic presentation of Figure 2 shows the different parts of that laboratory setup. The canal has been divided into two pools: the first one characterised by its small dimensions, named here “upstream pool”, acting in this case as an upstream reservoir; the second pool “downstream pool”, their dimension are more important compared to the one of the upstream pool, and it plays the role of the main pool to be automated.

The before mentioned pools are separated by a motorized upstream slide gate. Moreover, the extremity end of the downstream pool is equipped by a second gate manually adjustable. As it is shown in Figure 2, an instrumental platform equipped with ultrasonic level sensors (ULS) is installed to allow the real-time recording of experimental data with a sampling period equal to 0.15 s. The prototype is equipped also with a DC motor, a gate position sensor (GPS), a flow sensor, a speed variator and an electric pump. The canal is automated by using a programmable logic controller (PLC) via a control station based on a personal computer (PC) and with a SCADA (data acquisition and supervisory system).

In order to improve the performances of our prototype, in term of precision and rapidity, the SIMATICS7-300 is replaced in this work by another SIMATICS7-1500 characterised by its fast backplane bus, shortest reaction times, and a command processing time of up to 1 ns in the central processing unit (CPU). The water flows continually from the upstream to a downstream reservoir, and then it is pumped back to the upstream reservoir by the electric pump, whose frequency can be adjusted from 0 to 50 Hz. The principal measured signals are the upstream gate position $x_{up}\left(t\right)$, the upstream pool water level $y_{up}\left(t\right)$ and the upstream and downstream water levels of the main pool, $y_{dw}\left(t\right)$ and $y_{dwe}\left(t\right)$ respectively. A grid at the bottom of the upstream pool (where the water propelled by the pump enters the pool) uniformly distributes the flow through the entire pool and guarantees an approximately constant water surface level in all the pool ($y_{up}$ is approximately equal in all the pool).

Control of this hydraulic canal has some drawbacks because, due to the small volume of the upstream pool, the maneuvers of opening and closing the upstream gate produce big variations in the upstream water level. This problem is solved by implementing a control loop of the upstream water level, in order to keep its value in a fixed reference. A PID controller is used, that acts on the variable speed pump. This control law somehow filters the upstream water level variations, supporting the assumption that this level is approximately constant, which is used in the control of the downstream water level of the main pool.

## 3. Dynamic Models

### 3.1. Saint-Venat Equations

As it was mentioned in the Introduction, the Saint-Venant equations are regarded by the hydraulic engineers as an efficient way of modeling the dynamics of hydraulic canal systems. These are partial differential equations and are characterized by its nonlinearity. They express the conservation of momentum and mass of a one-dimensional canal flow. The following hypotheses are considered when deriving these equations, which apply to our hydraulic canal system:

- The considered flow is one-dimensional, the velocity is uniform, and the water level is horizontal.
- Vertical accelerations are negligible, and the pressure is hydrostatic.
- The average canal bed slope is small.
- The variation of canal width along the horizontal axis is small.

Denote the longitudinal abscissa by *x*, time by *t*, the wetted area by a(x,t) in $\left(m^{2}\right)$, the flow by q(x,t) in $\left(m^{3}/s\right)$, the velocity by v(x,t) in (m/s), the water level by y(x,t) in (m), the bed slope by $s_{b}\left(x\right)$ in (m/m), the friction slope by $s_{f}\left(x,t\right)$ in (m/m) and *g* the gravity constant. Then the Saint-Venant equations are:

(1) $$\frac{\partial a(x,t))}{\partial t}+\frac{\partial q(x,t))}{\partial x}=0$$

(2) $$\frac{\partial q(x,t))}{\partial t}+\frac{\partial }{\partial x}\left[\frac{q^{2}\left(x,t\right)}{a(x,t)}\right]+g\cdot a\left(x,t\right)\cdot \left(\frac{\partial y(x,t)}{\partial x}+s_{f}\left(x,t\right)-s_{b}\left(x\right)\right)$$

where (1) and (2) are the mass and momentum conservation equations respectively [6].

### 3.2. Linear Models of Integer Order

Saint-Venant equations are often linearized around flow regimes in order to develop appropriate control systems. The parameters of these resulting linear differential equations usually change in function of the operating flow regime. The nominal parameters and their ranges of variation are therefore estimated in order to include all the possible dynamics in an overall model. In order to obtain such model, incremental step tests are carried out around each possible flow regime. These tests allow to obtain the transfer function that relates the command for the upstream gate opening u(t) with the downstream water level of the main pool $y_{dwe}\left(t\right)$ (see Figure 2).

Delayed first-order models that result from linearizing around a specified operating regime are often used to describe these dynamics [31]:

(3) $$T_{i}\cdot D\Delta y_{dwe}\left(t\right)+\Delta y_{dwe}\left(t\right)=K_{i}\cdot \Delta u\left(t-L_{i}\right)$$

where D is the derivative operator, $K_{i}$ is the static gain, $T_{i}$ is the time constant and $L_{i}$ is the time delay. This kind of models will be denoted hereafter as *LIOM* (linear integer order models), and will be represented by the transfer function:

(4) $$G_{i}\left(s\right)=\frac{\Delta Y_{dwe}\left(s\right)}{\Delta U(s)}=\frac{K_{i}}{1+T_{i}\cdot s}e^{-L_{i}\cdot s}$$

Using a robust recursive parameters estimation and model validation method, a delayed second-order model was developed in [32] to accurately approximate the dynamic behavior of a real main irrigation canal. Such model precisely reproduced the dynamics of the considered irrigation canal even with data that had not been used in the fitting procedure, and in spite of some unmodeled dynamics. It is represented by the transfer function

(5) $$G_{s}\left(s\right)=\frac{\Delta Y_{dwe}\left(s\right)}{\Delta U(s)}=\frac{K_{s}}{\left(T_{1s}\cdot s+1\right)\left(T_{2s}\cdot s+1\right)}e^{-L_{s}\cdot s}$$

### 3.3. Linear Model of Fractional Order

In the last two decades, fractional-order calculus has emerged as a powerful tool to model processes described by partial differential equations, e.g., [21]. In the last years, this technique has been applied to model the dynamics of hydraulic canal systems. The first fractional-order model of an hydraulic canal was provided in [24]. This paper showed that the accuracy of a linear model could be improved by using a fractional-order model instead of an integer-order model. However, the comparison was carried out between a relatively simple integer-order model and a quite complex fractional-order model, leaving the question of whether it would be possible to obtain more accurate results with fractional-order models of similar complexity as integer-order models open. This question was somehow solved later in [25], in which a simple fractional-order model whose differential equation is

(6) $$T_{f}\cdot D^{\lambda_{f}}\Delta y_{dwe}\left(t\right)+\Delta y_{dwe}\left(t\right)=K_{f}\cdot \Delta u\left(t-L_{f}\right)$$

where $D^{\lambda_{f}}$ is the fractional derivative operator of order $\lambda_{f}$, and its transfer function is

(7) $$G_{f}\left(s\right)=\frac{\Delta Y_{dwe}\left(s\right)}{\Delta U(s)}=\frac{K_{f}}{1+T_{f}\cdot s^{\lambda_{f}}}e^{-L_{f}\cdot s}$$

significantly outperformed the accuracy of models (4) and (5), i.e., the fitting error between the response of the identified transfer function and the recorded data was reduced in more than 17%. This kind of models will be denoted hereafter *LFOM* (linear fractional order models). This model was later used to design a fractional-order control system for hydraulic canals [29].

Transfer functions like (7) were later used in [27] to model the dynamics of the two adjacent pools of the laboratory canal described in Section 2. This paper proposed a multivariable fractional-order system with two inputs (the frequency of the pump and the upstream gate opening command u(t)) and two outputs (the water levels of the first pool $y_{up}\left(t\right)$ and the downstream end of the main pool $y_{dwe}\left(t\right)$) (refer again to Figure 2). Such model significantly improved the description of the canal dynamics, reducing in more than 30% the ISE (integral squared error) between the model response and the recorded experimental data.

The above multivariable model was used later in [28] to improve the control system which is in charge of the water level of the first pool $y_{up}\left(t\right)$. By closing this loop, the two input/two output system of [27] becomes a single input/single output system whose input is u(t) and its output $y_{dwe}\left(t\right)$. The resulting transfer function is very complex and combines time delays with fractional-order derivatives. However, it was shown that the accuracy obtained with that transfer function in reproducing the canal dynamics is much better than the one obtained using models (4) or (5): the error index ISE was reduced in more than 50%.

### 3.4. Introducing Some Nonlinearity

It has been demonstrated that the modelization of a hydraulic canal system principally depends on its geometry and the flow characteristics [6]. Hydraulic canal systems can present two types of flow. The first type is denoted *free flow* and is verified if the flow at the canal is critical or supercritical. In such a case, the downstream level water has no influence on the flow and the water discharge depends only on the upstream water level. Our canal works in a free flow regime if the following condition is verified [33]:

(8) $$y_{up}>0.81\cdot y_{dwe}\cdot {\left(\frac{y_{dwe}}{x_{up}}\right)}^{0.72}$$

where $x_{up}\left(t\right)$ is the upstream gate opening, whose value is very close to the command signal u(t).

The discharge through the gate is approximated in this case by:

(9) $$q_{f}=c_{d}\cdot b\cdot x_{up}\cdot {\left(2\cdot g\cdot y_{up}\right)}^{0.5}$$

where $q_{f}$ is the flow through the gate, *b* is the width of the upstream gate and $c_{d}$ is a discharge coefficient, generally close to 0.6.

The second type of flow is denoted *submerged flow*. A hydraulic canal system works in a regime of submerged flow if the flow remains subcritical at the canal. In that case, the flow is influenced by the downstream level. Our canal works in a submerged flow regime if the following condition is verified [33]:

(10) $$y_{dwe}<y_{up}<0.81\cdot y_{dwe}\cdot {\left(\frac{y_{dwe}}{x_{up}}\right)}^{0.72}$$

The discharge through the gate is approximated in this case by:

(11) $$q_{s}=c_{d}\cdot b\cdot x_{up}\cdot {\left(2\cdot g\cdot \left(y_{up}-y_{dwe}\right)\right)}^{0.5}$$

### 3.5. Dynamic Models Including the Nonlinearity

The combination of the static nonlinear models of Section 3.4 with the dynamic models described in Section 3.2 and Section 3.3 yields the following nonlinear dynamic models.

In the case of free flow, two models will be considered: an integer order model

(12) $$T_{i}\cdot D\Delta y_{dwe}\left(t\right)+\Delta y_{dwe}\left(t\right)=K_{i}\cdot {\left(y_{up}\left(t\right)\right)}^{\gamma_{i}}\cdot \Delta u\left(t-L_{i}\right)$$

denoted hereafter *NLIOM-F* (nonlinear integer order models under free flow) and a fractional order model

(13) $$T_{f}\cdot D^{\lambda_{f}}\Delta y_{dwe}\left(t\right)+\Delta y_{dwe}\left(t\right)=K_{f}\cdot {\left(y_{up}\left(t\right)\right)}^{\gamma_{f}}\cdot \Delta u\left(t-L_{f}\right)$$

denoted hereafter *NLFOM-F* (nonlinear fractional order models under free flow). In both models, the power of the flow expression (9) has been generalized to a real number γ. In the standard case considered so far, γ is made equal to 0.5 in (12) and (13).

Other two models will be considered in the case of submerged flow: an integer order model

(14) $$T_{i}\cdot D\Delta y_{dwe}\left(t\right)+\Delta y_{dwe}\left(t\right)=K_{i}\cdot {\left(y_{up}\left(t\right)-y_{dwe}\left(t\right)\right)}^{\gamma_{i}}\cdot \Delta u\left(t-L_{i}\right)$$

denoted hereafter *NLIOM-S* (nonlinear integer order models under submerged flow) and a fractional order model

(15) $$T_{f}\cdot D^{\lambda_{f}}\Delta y_{dwe}\left(t\right)+\Delta y_{dwe}\left(t\right)=K_{f}\cdot {\left(y_{up}\left(t\right)-y_{dwe}\left(t\right)\right)}^{\gamma_{f}}\cdot \Delta u\left(t-L_{f}\right)$$

denoted hereafter *NLFOM-S* (nonlinear fractional order models under submerged flow). In both models, the power of the flow expression (11) has also been generalized to a real number γ. The standard case considered so far corresponds to a value γ=0.5 in (14) and (15).

## 4. Identification Technique

Models *LIOM* and *LFOM* assume that the upstream water level is constant. Since our first pool is of relatively reduced volume, this condition is achieved by closing the already mentioned loop around the pump and the first pool, so that the water level $y_{up}\left(t\right)$ is controlled. The nonlinear models (12)–(15) take into account variations in $y_{up}\left(t\right)$, i.e., the state of the adjacent upstream pool. Therefore, these models do not need that $y_{up}\left(t\right)$ be constant and the control of the water level $y_{up}\left(t\right)$ neither.

The aim of this work is to obtain an accurate model of the main pool of our laboratory canal. The input of this model is the command to the gate opening u(t) and the output is the downstream water level $y_{dwe}\left(t\right)$. An identification procedure is proposed in the time domain in which different dynamic models will be identified from the time responses obtained exciting the system with pseudo-random binary signals (hereafter denoted PRBS signals). A question is how to carry out the identification of fractional order models using time responses. The technique used here is based on the method proposed in [34] to identify fractional-order dynamics in electrochemical processes. It applied pulse inputs and used the Grünwald-Letnikov definition of a fractional-order derivative to numerically simulate fractional-order ordinary differential equations Appendix A [21]. Moreover, a main concern about our identification procedure is how to handle the nonlinearity associated to the gate movements and the quantization of the output data caused by our ultrasonic sensors.

The proposed identification methodology takes into account the above issues and is composed of the following steps:

1. Define an operating flow regime.
2. Apply an incremental step command to u(t), record the output $y_{dwe}\left(t\right)$ and obtain a preliminary approximate simplified model.
3. Check the above mentioned phenomena of the nonlinear gate movement and output measurement quantization. If necessary, include these phenomena in the dynamic model to be obtained.
4. Design the PRBS from the information about the process obtained in step 2.
5. Excite the system with the designed PRBS, which is applied incrementally over the operating point at u(t). Record the process output $y_{dwe}\left(t\right)$ and carry out the identification of the parameters of the proposed model from this data.

The last step of the procedure will be carried out for all the proposed linear models LIOM and LFOM, and nonlinear models NLIOM-*F*, NLFOM-*F*, NLIOM-*S* and NLFOM-*S*. Moreover, all this procedure will be repeated at different flow regimes with the aim of obtaining a global dynamic model of the main pool of our canal. Details about some of the above mentioned steps are given next.

### 4.1. Step Responses

Time domain experiments based on step input signals have been carried out in the considered hydraulic canal. The principal aim is to develop initial models that describe the canal behavior around several operating flow regimes. During these experiments, the water level of the upstream pool was maintained constant and equal to 60 mm, the downstream gate was maintained constant and the upstream gate realized several step movements with the purpose of exciting the canal dynamics in different operating regimes. All the signals involved in the process were measured with a sampling time h=0.13 s. In these experiments, the input was u(t) and the output $y_{dwe}\left(t\right)$.

In these experiments, the control of the upstream pool water level $y_{up}\left(t\right)$ by varying the pump frequency was closed. That pump is configured for a range of frequency variation from 0 to 50 Hz. Then $y_{up}\left(t\right)$ was assumed to be 60 mm in all the experiments. Similar experiments with similar results were carried out in [29]. Then we refer to the results and dynamic models reported in that paper. They show that the maximum time constant of such models (obtained for different flow regimes) is $T_{max}=2.86$ s, which yields a maximum settling time $T_{s,max}=14.32$ s. Additionally, we have carried out here an analysis of the frequency responses obtained with these models. It showed that the fastest among these processes had a bandwidth (frequency at which the magnitude of the Bode plot drops below 3 dB of the process gain) of $f_{max}=0.88$ Hz. This value defines the minimum bandwidth of the spectrum of the input signal required to identify all the linearized models of the process.

### 4.2. Nonlinear Gate Behaviour and Measurement Quantization

**Figure 3:**
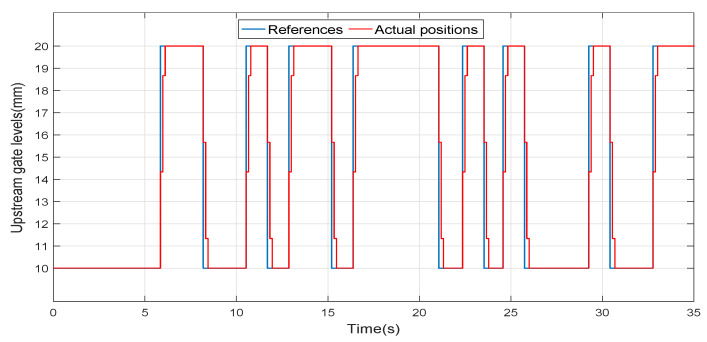
Upstream gate movements.

Figure 3 shows a typical movement of the upstream gate in our experiments. A difference can be observed between the control signal u(t), which is the variable that we can manipulate, and is the reference for the servopositioning system of the gate, and the actual gate opening $x_{up}\left(t\right)$. This difference is caused because the positioning system of the gate moves at a constant speed until the target position is reached, instant at which the gate stops. This nonlinear behaviour can be usually ignored in step input experiments in which only the slow dynamic components of the process have to be characterized. However, our purpose when exciting the system with PRBS is to characterize also the fast dynamic components and, in this case, this nonlinear behaviour may alter the accuracy of the identification process.

The nonlinear model proposed here for the gate movement is

(16) $${\dot{x}}_{up}\left(t\right)=\left\{\begin{array}{cc} \nu \cdot sign\left(u\left(t\right)-x_{up}\left(t\right)\right), & \mathrm{if}\;x_{up}\left(t\right)\neq u\left(t\right)\\ 0, & \mathrm{if}\;x_{up}\left(t\right)=u\left(t\right) \end{array}\right.$$

where ν represents the velocity of the gate.

In our canal, water levels are measured using ultrasonic sensors whose resolution is 0.05 mm. In order to take this into account in the identification process, the quantization of these signals is also included in the fitted models. This phenomenon is represented by

(17) $${\hat{y}}_{dwe}\left(t\right)=r\cdot nint\left(\frac{y_{dwe}\left(t\right)}{r}\right)$$

where ${\hat{y}}_{dwe}\left(t\right)$ is the real data resulting from the quantization process, r=0.05 is the sensor’s resolution and nint(x) is the nearest integer value function.

### 4.3. PRBS Design

Frequency domain analysis requires principally a suitable excitation signal. The use of random signals has become widespread and often replaces the standard sine technique, that could sometimes be laborious. The characteristics of the excitation signal are often derived from the features of the system to be identified and the desired method of signal processing, being a goal that these signals approximate as much as possible the white noise signal characteristics.

White noise spectrum has the interesting feature of having a flat spectrum, i.e., a spectrum with infinite bandwidth. This makes white noise signals very appropriate excitation signals for identification processes. The spectrum of PRBS accurately approximates the white noise spectrum in a certain frequency band. This feature makes PRBS be often used as excitation signals in identification techniques.

#### 4.3.1. PRBS Generation

The utilization of PRBS requires a strictly correct design of its parameters in order to excite the system in all the frequency range in which the system dynamics is relevant. PRBS generators can be realized by shift registers with module 2 (XOR) feedback at predetermined “tap” positions [35]. A look-up table can be used within an embedded environment to generate this periodic signal. If *n* represents the number of stages, the number of terms of the sequence to be repeated is $N=2^{n}-1$. If Δt s were the period of the clock of the PRBS generator, the period of the generated sequence would be T=N·Δt s. This period *T* is a critical parameter of the PRBS because its value must be chosen so that the impulse response of the system to be identified should had become nearly null once this time has elapsed.

#### 4.3.2. Power Spectrum Bandwidth of the PRBS

Since the Wiener–Khintchine theorem states that the power spectral density of a wide sense stationary random process is the Fourier transform of the corresponding autocorrelation function, it can be proven that the PRBS has a power spectrum whose magnitude envelope is described by the function sinc(x). This magnitude reaches zero for the first time at the clock angular velocity $\frac{2\cdot \pi }{\Delta t}$ rad/s [35]. Then the PRBS bandwidth is lower than the frequency of the clock, having in practice a usable frequency range of 1/3 of the clock frequency. If $f_{max}$ is the maximum frequency of interest, a general rule of thumb is to choose the clock frequency $f_{c}=1/\Delta t$ to be approximately

(18) $$f_{c}=2.5f_{max}$$

The frequencies of the spectrum are discrete with a period $\Delta \omega =\frac{2\cdot \pi }{N\cdot \Delta t}$, which is defined by the number of terms of the sequence *N* and the clock period Δt.

The complete sequence of the PRBS must be executed in order to keep its white-noise-like behavior. The design of the PRBS should consider the frequency bandwidth of interest and the duration of the test.

The sampling rate is dictated by $f_{c}$. Since the Nyquist–Shannon sampling theorem must be taken into account, a sampling a rate of 2–5 times $f_{c}$ must be used. Taking into account that our sampling period is h=0.13 s, the clock period Δt is chosen to be 0.5 s (about four times *h*), in accordance with the previous consideration. Note that $f_{c}=2$ Hz in this case, which approximately verifies (18): 2 Hz ≈ 2.5 × 0.88 Hz.

The responses to step commands obtained in [29] show a maximum time delay of 5.2 s and a maximum time constant of about 3 s. Then the maximum settling time is about 14 s. In order to guarantee that the impulse response becomes zero (or the step response reaches its steady state) in the interval of execution of the PRBS, a value n=9 has been chosen, which implies N=511 and T=2555 s.

**Figure 4:**
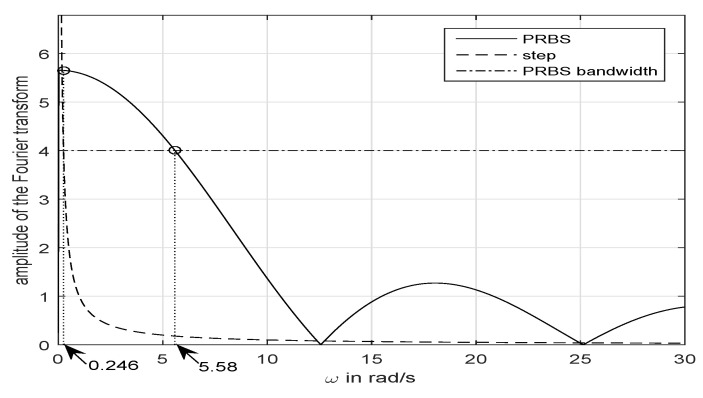
Magnitude of the Fourier transforms of a PRBS input and a step input that have the same amplitudes.

Figure 4 explains why PRBS signals are a more appropriate tool to identify systems than the fundamental step signal. The magnitude of the Fourier transforms (hereinafter denoted MFR) of a unity step input and our designed PRBS input with unity amplitude are shown in this figure. We consider that the bandwidth of the MFR of the PRBS is defined by a decrease of 3 dB with respect to its maximum value, i.e., $1/\sqrt{2}$ of the maximum value. This figure shows that the amplitude that defines the bandwidth is 4, and the value of the resulting bandwidth is 5.58 rad/s. However the range of frequencies at which the MFR of the unity step input is over 4 is only [0,0.246]. This means that our PRBS input significantly excites the dynamics of our canal pool in a frequency range more than 20 times higher than the step input of the same amplitude. Then we foresee to characterize the high frequency dynamics of our canal more accurately using PRBS inputs than in our previous experiments in which we used step inputs.

## 5. Experimental Data and Fitting Indexes

**Figure 5:**
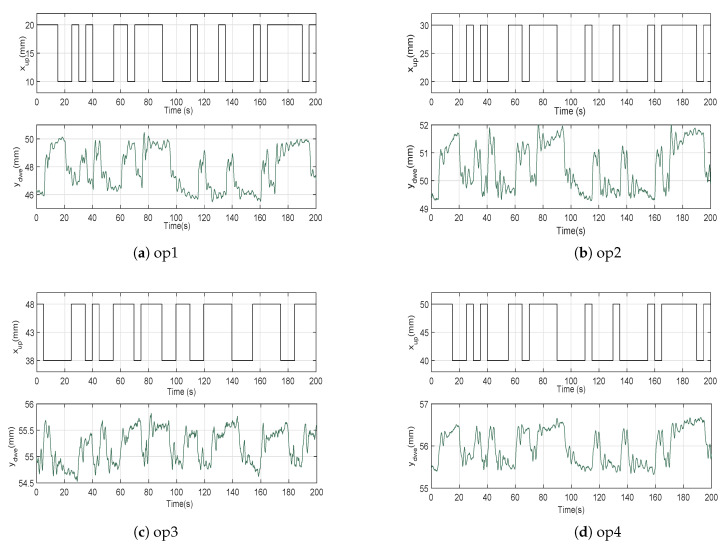
Operating points considered for the identification procedure.

The PRBS designed in the previous section is applied with an amplitude of ±5 mm to generate upstream gate opening incremental commands. In Figure 5, the data recorded in our identification procedure is shown for four operating points. These operating points are defined by gate openings of 15 mm (op1), 25 mm (op2), 43 mm (op3) and 45 mm (op4), which determine the different ranges of the upstream gate movements. The control system connected to the electric pump maintains the water level of the first pool at an approximate value of 60 mm during all the experiments.

Models introduced in Section 3 were tested on the validation data of Figure 5. The normalized root mean-squared error (hereafter denoted Nrmse) is used as a measure of the accuracy provided by the model fitted to the data. This criterion is a non-dimensional version of the root-mean-squared error RMSE and is given by the expression [36]:

(19) $$Nrmse=100\cdot \left(1-\frac{{∥{\bar{y}}_{dwe}\left(t\right)-y_{dwe}\left(t\right)∥}_{2}}{{∥y_{dwe}\left(t\right)-mean\left(y_{dwe}\left(t\right)\right)∥}_{2}}\right)$$

where ${\bar{y}}_{dwe}\left(t\right)$ is the response of the model and $y_{dwe}\left(t\right)$ is the real data. Nrmse allows to compare data of different dimensions dividing the RMSE by the range of the observed data. The tested model is a perfect fit if the cost criterium (19) applied to the validation data is 100%. Lower values indicate a decreasing fit. In this study we consider that only cost values over 70% correspond to acceptable fittings [37]. Moreover, the Nrmse index will be used to compare the accuracy of the proposed models.

In order to carry out the parameter identification procedure, a recursive parameters estimation algorithm was executed, which is based on the minimization of the Integral Absolute Error (IAE):

(20) $$IAE_{j}=\int_{0}^{\infty }\left|{\bar{y}}_{dwe}\left(t\right)-y_{dwe_{j}}\left(t\right)\right|\cdot dt$$

where $y_{dwe_{j}}\left(t\right)$ is the response obtained in the operating regime op*j*, with 1≤j≤4. The results of such identification process were evaluated using the Nrmse index (19).

We use in the identification task a different cost (20) than in the validation task (19) because this last index tends to ignore small errors (it squares the errors and, then, small errors produce an even smaller value when squared to be included in the cost) while we want that our identified models reproduce as close as possible the small oscillations that appear in the responses of Figure 5.

We are also interested in identifying a single model that is able of accurately reproduce the dynamic behaviour in the four operating regimes of Figure 5. In this case, the parameter identification procedure uses a recursive parameters estimation algorithm based on the minimization of the combined Integral Absolute Error ($IAE_{c}$) of the four responses:

(21) $$IAE_{c}=\sum_{j=1}^{4}IAE_{j}=\sum_{j=1}^{4}\int_{0}^{\infty }\left|{\bar{y}}_{dwe}\left(t\right)-y_{dwe_{j}}\left(t\right)\right|\cdot dt$$

## 6. Fitting the Linearized Models

LIOM and LFOM models were fitted to the data presented in Figure 5. These models were completed including the nonlinear effects (16) and (17).

### 6.1. Integer Order Models

The four responses of Figure 5 were approximated by their respective LIOM models. The time delay $L_{i}$, time constant $T_{i}$ and static gain $K_{i}$ were optimized for the operating regimes 1≤j≤4 minimizing the corresponding $IAE_{j}$ indexes, as it was explained in the previous section. These identified parameters are shown in Table 1. The corresponding Nrmse indexes are shown in the last column. It can be observed that this model does not reproduce adequately the behaviour around the operating regimes op2 and op3 (their Nrmse are lower than 70%).

Subsequently, a *nominal model* is determined that attempts to reproduce the behaviour of the four operating points. In this case, the parameters of the LIOM model are estimated minimizing the $IAE_{c}$ index of expression (21). The last column of Table 1 indicates that this single model is unable to reproduce adequately the dynamic behaviour at any of the four operating regimes (all the Nrmse are below the 70%). The last row of Table 1 shows the maximum relative deviation of the parameters identified minimizing the $IAE_{j}$ indexes with respect to the parameters obtained minimizing the $IAE_{c}$ index:

(22) $$\Delta p=max_{1\leq j\leq 4}\frac{\left|p_{j}-p_{c}\right|}{\left|p_{c}\right|}$$

where *p* is a generic parameter that was first identified in each operating regime ($p_{j}$) (using (20)) and later identified taking jointly into account all the responses ($p_{c}$) (using (21)).

### 6.2. Fractional Order Models

The four responses of Figure 5 were approximated now by their respective LFOM models. The time delay $L_{f}$, time constant $T_{f}$, fractional order derivative $\lambda_{f}$ and static gain $K_{f}$ were optimized again for the operating regimes 1≤j≤4 minimizing the corresponding $IAE_{j}$ indexes. These identified parameters are shown in Table 2. The corresponding Nrmse indexes are shown in the last column. In this case, this model reproduces adequately the behaviour around all the operating regimes because all the Nrmse are higher than 70%. Moreover, in all the cases, the Nrmse indexes yielded by the LFOM models are higher than the ones yielded by the previous LIOM models.

Subsequently, a nominal model is determined that attempts to reproduce the behaviour of the four operating points. The parameters of the LFOM model are estimated minimizing the $IAE_{c}$ index of expression (21). The last column of Table 2 indicates that this single model is again unable to reproduce adequately the dynamic behaviour of all the four operating regimes. However, in this case, it only fails in reproducing op2 and op3 with the required accuracy. The last row of Table 2 shows again the maximum relative deviation of the parameters identified minimizing the $IAE_{j}$ indexes with respect to the parameters obtained minimizing the $IAE_{c}$ index, according to expression (22).

## 7. Fitting of Partially Non-Linear Models

### 7.1. Checking the Flow Type Conditions

**Figure 6:**
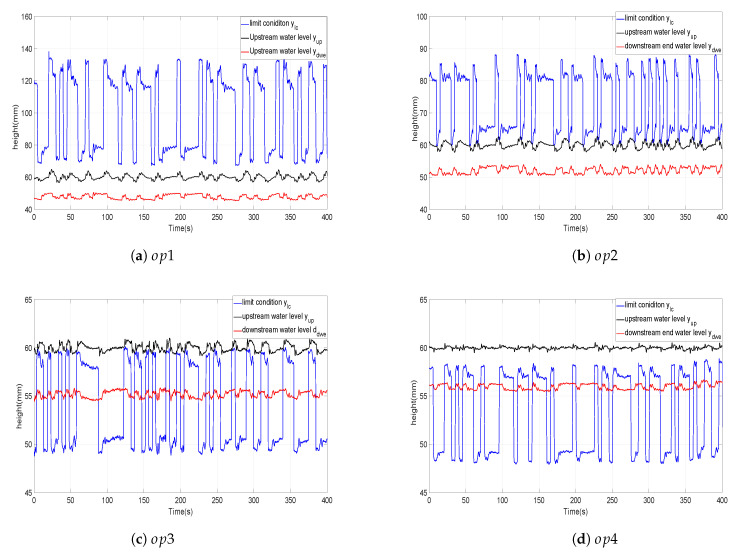
Flow conditions verified with the defined operating regimes.

In Section 3, the submerged and free types of flow were defined. Moreover, conditions that allow to identify these flows were given by expressions (8) and (10) [33]. Figure 6 shows the evolution of the downstream end water level $y_{dwe}\left(t\right)$, the upstream water level $y_{up}\left(t\right)$ and the limit condition between free and submerged flows:

(23) $$y_{lc}\left(t\right)=0.81y_{dwe}\left(t\right){\left(\frac{y_{dwe}\left(t\right)}{x_{up}\left(t\right)}\right)}^{0.72}$$

Figure 6a,b respectively show that the manoeuvres around regimes op1 and op2 are always of submerged flow type since condition (10) is always verified in both cases ($y_{lc}\left(t\right)>y_{up}\left(t\right)>y_{dwe}\left(t\right)$). Conversely, Figure 6c,d shows that the manoeuvres around regimes op3 and op4 are always of free flow type since condition (8) is always verified ($y_{lc}\left(t\right)<y_{up}\left(t\right)$).

Considering the results of Figure 6b, in which the border of the submerged flow condition is reached at some instants (sometimes $y_{lc}\left(t\right)=y_{up}\left(t\right)$) that basically coincide with a gate opening of 30 mm, we can advance the conjecture that submerged flow is achieved for upstream gate openings lower than $x_{up,lim1}=30$ mm. It is mentioned that the opening range is [0,50] mm in the upstream gate of our prototype.

In another part, according to Figure 6c, it is possible to note that the border of the free flow condition is reached at some instants (sometimes $y_{lc}\left(t\right)=y_{up}\left(t\right)$), which coincide with an upstream gate opening of 38 mm. we can advance the conjecture that free flow is achieved for upstream gate openings higher than $x_{up,lim2}=38$ mm.

**Figure 7:**
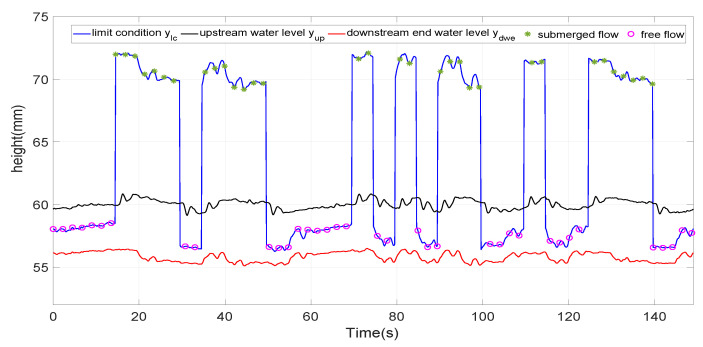
Flow types of the operating point op5.

Thereafter, the interval [30,38] is defined as the border interval between the two types of flow when a PRBS with such characteristics is considered. Other way, defining step movement that reaches the before mentioned interval yields to a mixing flow condition as it was detailed in Figure 7, where the data corresponding to a fifth defined operating point $op_{5}$ defined by gate opening of 35 mm. The manoeuvres around regimes op5 are sometimes of free flow type (it is verified that $y_{lc}\left(t\right)<y_{up}\left(t\right)$) and sometimes of submerged flow type (it is verified that $y_{lc}\left(t\right)>y_{up}\left(t\right)>y_{dwe}\left(t\right)$).

### 7.2. Submerged Flow Regime

#### 7.2.1. Identification

The nonlinear models NLIOM-*S* and NLFOM-*S* are fitted to the data of Figure 5a,b. The parameters of these models are again obtained using a recursive parameters estimation algorithm based on the minimization of the Integral Absolute Error. Such algorithm is based on the robust ’Trust Region reflective technic’ presented to demonstrate the trust region approaches. The fractional-order calculus based toolbox ’FOMCON’ is considered when defining the fractional-order of the developed models [38]. First, We will estimate different models for the operating regimes op1 and op2, minimizing indexes $IAE_{1}$ and $IAE_{2}$ respectively. Later a single model for all the submerged flow type regimes will be obtained minimizing the combined index of the submerged flow cases op1 and op2: $IAE_{cs}=IAE_{1}+IAE_{2}$.

In the scientific literature, γ is considered equal to 0.5. However, we will include the parameter γ in the optimization procedure in order to assess if values of γ different from 0.5 would improve the accuracy of the identified models. A range of variation 0≤γ≤10 was considered. Then, the previous identification procedure has been modified, and now consists of the following steps: (1) given a value of γ, minimize the $IAE_{j}$, (2) obtain the Nrmse value with the estimated parameters, (3) repeat steps 1 and 2 for all the range of values of γ and (4) choose the value of γ that yields the maximum Nrmse.

**Figure 8:**
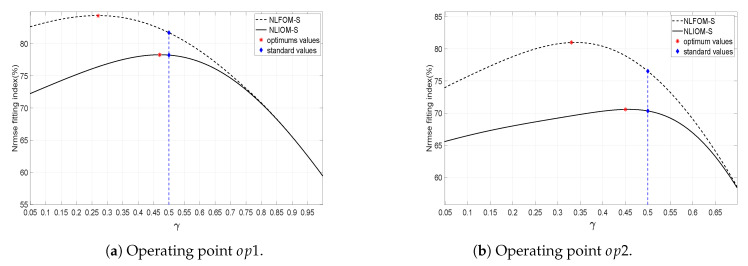
Evolution of the fitting index Nrmse in function of γ.

The evolution of the Nrmse index in function of γ is shown in Figure 8a,b for regimes op1 and op2 respectively. Plots of the cost functions in the cases of the two models NLIOM-*S* and NLFOM-*S* are shown in these figures. The optimum values are also marked in these plots.

Table 3 and Table 4 show the parameters obtained from the previous identification procedure in the cases of the integer and fractional order models: the time delays $L_{i}$ and $L_{f}$, the time constants $T_{i}$ and $T_{f}$, the fractional order derivative $\lambda_{f}$ and the static gains $K_{i}$ and $K_{f}$ respectively. This process was carried out for the flow regimes op1 and op2. The corresponding Nrmse indexes are listed in the last column of these two tables. These values are higher than the ones obtained in Table 1 and Table 2. Comparing Table 1, Table 2, Table 3 and Table 4, it can be stated that: (1) the NLIOM-*S* model outperforms the LIOM model (moreover, regime op2 is adequately modelled by NLIOM-*S*, unlike what happened with LIOM), (2) the NLFOM-*S* model outperforms the LFOM model and (3) the NLFOM-*S* models outperform the NLIOM-*S* models (the Nrmse of the NLFOM-*S* models reach values even higher than 80%). The improvements between 5% and 10% achieved in the Nrmse as consequence of using the NLFOM-*S* model instead of the NLIOM-*S* model justify the use of fractional order models when our canal is operating in a submerged flow mode. 

Subsequently, a nominal model was determined that attempts to reproduce the behaviour of the two operating regimes op1 and op2. The parameters of the NLIOM-*S* and NLFOM-*S* nominal models are estimated minimizing the $IAE_{cs}$ index defined before, and are included in Table 3 and Table 4 together with their corresponding Nrmse. The last columns of Table 3 and Table 4 indicate that the NLIOM-*S* and NLFOM-*S* nominal models are able to reproduce adequately the dynamic behaviour of the two considered operating regimes. The last row of Table 3 and Table 4 show again the maximum relative deviation of the parameters identified minimizing the $IAE_{j}$ indexes with respect to the parameters obtained minimizing the $IAE_{cs}$ indexes, according to expression (22).

The results of Table 3 show that the only parameter that experiences relatively large variations when the operating regime changes is the time constant $T_{i}$ (about ±11%). Conversely, the time delay $L_{i}$, the static gain $K_{i}$, and the parameter $\gamma_{i}$ that characterizes the submerged flow operation experience slight variations which have little influence in the dynamics of the main canal pool. Table 4 shows also that the time delay $L_{f}$, the static gain $K_{f}$, and the parameter $\gamma_{f}$ experience slight variations with the changes in the operating regime. However, the time constant $T_{f}$ and the fractional order $\lambda_{f}$ experience relatively large variations (about ±12%). Comparing the last row of Table 1 with the last row of Table 3, i.e the linear and nonlinear integer order models, and the last row of Table 2 with the last row of Table 4, i.e., the linear and nonlinear fractional order models, it is observed that the range of variation of the parameters, i.e., the index (22), is reduced if the nonlinear models are used instead of the linear ones in both integer order and fractional order models.

#### 7.2.2. Validation

The NLIOM-*S* and NLFOM-*S* nominal models shown in Table 3 and Table 4 are compared to the LIOM and LFOM nominal models shown in Table 1 and Table 2. In this validation procedure, in order to assess the quality of the obtained models, we check if the responses provided by them are able to accurately reproduce some new data of the canal that has been recorded.

**Figure 9:**
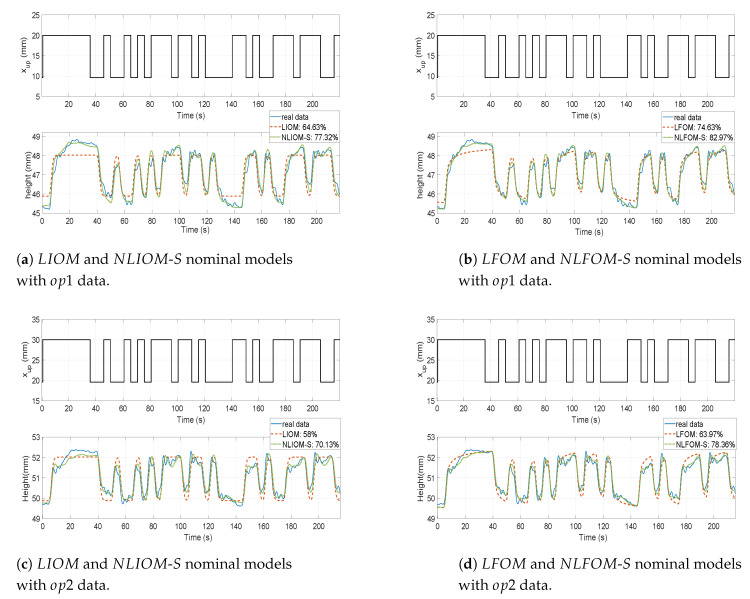
Validation of the obtained nominal models with op1 and op2 data.

Figure 9 shows the matching attained by all the proposed nominal models to the new data. In all the cases, a significant improvement is observed in the quality of the matching if nonlinear models are used instead of linear models.

Subsequently, data obtained from applying the PRBS input to other operating points of submerged flow type than op1 and op2 has been used to validate the models. These new operating points are defined by gate openings of 13 mm and 23 mm, and are denoted operating points $op1^{*}$ and $op2^{*}$ respectively. Table 5 shows the Nrmse matching indexes obtained using the four considered nominal models LIOM, LFOM, NLIOM-*S* and NLFOM-*S* in the cases of the four operating points that have been chosen inside the submerged flow region: [13;23] mm.

The results of this table show an improvement close to 15% when the NLIOM-*S* model is used instead of the LIOM model and an improvement close to 13% when the NLFOM-*S* model is used instead of the LFOM model. This table also shows that the best matchings (highest Nrmse indexes) have been provided by the NLFOM-*S* nominal model. Moreover, using this model, the Nrmse index is around the 80% in the four operating points, which indicates a very good matching in all the cases, and suggests that this model accurately reproduces the dynamics of our main pool dynamics in all the submerged flow cases. 

### 7.3. Free Flow Regime

#### 7.3.1. Identification

The nonlinear models NLIOM-*F* and NLFOM-*F* are fitted to the data of Figure 5c,d. The parameters of these models are again obtained using a recursive parameters estimation algorithm based on the minimization of the Integral Absolute Error. First we estimate different models for the operating regimes op3 and op4, minimizing indexes $IAE_{3}$ and $IAE_{4}$ respectively. Later a single model for all the free flow type regimes will be obtained minimizing the combined index of the free flow cases op3 and op4: $IAE_{cf}=IAE_{3}+IAE_{4}$.

**Figure 10:**
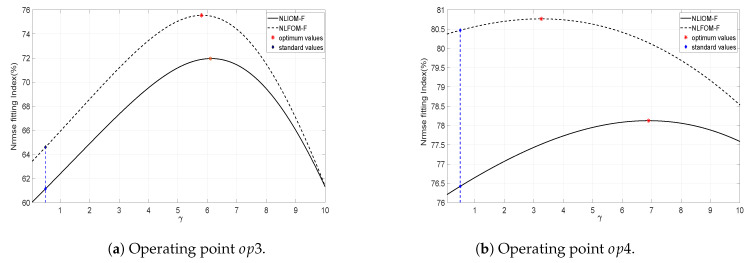
Evolution of the fitting index Nrmse in function of γ.

In this type of flow, the parameter γ of the models is also varied between 0 and 10 in order to obtain a better fitting of the experimental data. Then, the identification procedure described in the previous subsection is also used here. The evolution of the Nrmse index in function of γ is shown in Figure 10a,b for regimes op3 and op4 respectively. Plots of the cost functions in the cases of the two models NLIOM-*F* and NLFOM-*F* are shown in these figures. The optimum values are also marked in these plots. It is noted that optimizing γ in the interval [0, 10] yields an improvement with respect to the standard value of γ (which is 0.5) close to 10% when considering op3 data. Such improvement is less important in the case of op4 data. It is essential to note that, the value of γ has been obtained from a mathematical process, and it has no physical interpretation in our models.

Table 6 and Table 7 show the parameters obtained from the identification procedure in the cases of the integer and fractional order models: the time delays $L_{i}$ and $L_{f}$, the time constants $T_{i}$ and $T_{f}$, the fractional order derivative $\lambda_{f}$ and the static gains $K_{i}$ and $K_{f}$ respectively. This process was carried out for the flow regimes op3 and op4. The corresponding Nrmse indexes are listed in the last column of these two tables.

Subsequently, a nominal model was determined that attempted to reproduce the behaviour of the two operating regimes op3 and op4. The parameters of the NLIOM-*F* and NLFOM-*F* nominal models were estimated minimizing the $IAE_{cf}$ index defined before, and are included in Table 6 and Table 7 together with their corresponding Nrmse. The last column of Table 6 indicates that the nominal model NLIOM-*F* is unable to reproduce adequately the dynamics at op3. The time constant $T_{i}$ in the case NLIOM-*F* changes considerably which is justified by an uncertainty index of 38.77%. Such variation is caused by the numerical precision of the optimization procedure when finding the local minimization and by the search region of the algorithm, and it is not relevant since an average model is finally proposed.

Table 7 shows that, however, the nominal model NLFOM-*F* reproduces adequately the dynamic behaviour of op3 and op4. The last row of Table 6 and Table 7 show again the values of the parameter deviation index (22) when the parameters have been obtained from minimizing the $IAE_{cf}$ index.

Comparing Table 1, Table 2, Table 6 and Table 7, it can be stated that: (1) fitting NLIOM-*F* models instead of LIOM models to op3 or op4 data does not improve the Nrmse indexes, (2) the NLIOM-*F* nominal model outperforms the LIOM nominal model though the fitting index of the former one is still below 70% in the case of the op3, (3) the NLFOM-*F* model provides fitting indexes similar to the ones of the LFOM model, both in the cases of the models fitted to particular operating regimes and the nominal model and (4) the NLFOM-*F* (and LFOM) models outperform the NLIOM-*F* models, yielding Nrmse values over 70% in both cases of models, the models particularized to operating points and the nominal model. The improvements between 4% and 8% achieved in the Nrmse as consequence of using fractional order models instead of integer order models justify the use of the formers when our canal is operating in a free flow mode.

Figure 10b shows that in the case of op3 and op4 (the gate is very open), the optimized values of the parameter γ are clearly higher than the ones fitted with submerged flow data, i.e., the main pool dynamics is Importantly affected by the upstream pool water level $y_{up}\left(t\right)$.

#### 7.3.2. Validation

The NLIOM-*F* and NLFOM-*F* nominal models shown in Table 6 and Table 7 are compared to the LIOM and LFOM nominal models shown in Table 1 and Table 2. In this validation procedure, in order to assess the quality of the obtained models, we check if the responses provided by them are able to accurately reproduce some new data of the canal that has been recorded.

**Figure 11:**
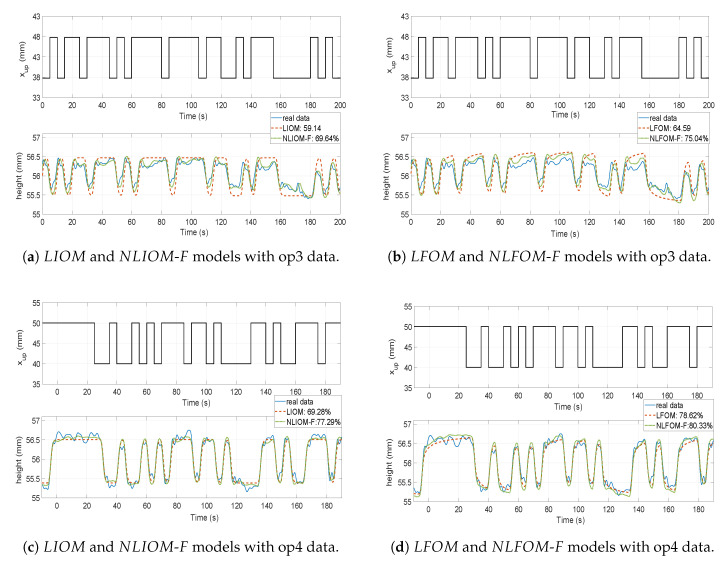
Validation of the obtained nominal models with op3 and op4 data.

Figure 11 shows the matching attained by all the proposed nominal models to the new data. It can be observed that a significant improvement is observed in the quality of the matching if nonlinear models are used instead of linear models in the fractional order case. However, differences can be barely observed between the responses of nonlinear and linear models in the integer order case.

Subsequently, data obtained from applying the PRBS input to other operating points of free flow type than op3 and op4 has been used to validate the models. These new operating points are defined by gate openings of 43 mm and 45 mm, and are denoted operating points $op3^{*}$ and $op4^{*}$ respectively. Table 8 shows the Nrmse matching indexes obtained using the four considered nominal models LIOM, LFOM, NLIOM-*F* and NLFOM-*F* in the cases of the two operating points that have been chosen inside the free flow region: [43;45] mm.

The results of this table show an improvement close to 5% when the NLIOM-*F* model is used instead of the LIOM model and an improvement close to 4% when the NLFOM-*F* model is used instead of the LFOM model. This table also shows that the best matchings (highest Nrmse indexes) have been provided by the NLFOM-*F* nominal model. Moreover, using this model, the Nrmse index is around the 75% in the four operating points, which indicates a good matching in all the cases, and suggests that this model accurately reproduces the dynamics of our main pool dynamics in all the free flow cases.

## 8. Conclusions

This paper has addressed the modelization of the nonlinear dynamics of a laboratory hydraulic canal installed in the University of Castilla-La Mancha. Improving the precision of its dynamic model allows for a best design of the automatic control system of the canal, which is in charge of delivering the desired water flow at its downstream end. Since the structure of the dynamic model as well as the values of the model parameters highly change in function of the operating flow regimes, obtaining a relatively simple dynamic model that reproduces all the observed complex dynamics is a difficult task.In order to design a subsequent control system, a compromise has to be reached between the complexity of the process model (it should be as simple as possible in order to facilitate the tasks of the analysis and design of the control system) and the accuracy in replicating the canal dynamics.

Previous papers on identifying the dynamics of this system [25,27,28,29] reported linear models—of either integer or fractional order natures—that were fitted to responses of the canal to step commands. These models were identified around a specific operating point and badly reproduced the canal behaviour when the operating point diverged from that specific one. Moreover, we noticed some high frequency dynamics (as for example small high frequency oscillations) in the responses of the canal that could not be efficiently characterized by using simple step commands since these do not excite the high frequency dynamics of the process. Then we have excited the main pool of the canal with specially designed pseudo-random binary signals, in order to characterize also its high frequency behaviour.

Several models have been tested of integer and fractional orders combined with linear and nonlinear structures. Actuator and sensor nonlinearities, i.e, the manoeuvres of the gate (16) and the quantization of the measurements (17) have been considered. Comparison of the results of some identification tests carried out using the command signal u(t) or the real gate opening $x_{up}\left(t\right)$ as inputs revealed that the differences in the parameters estimated using either input were insignificant. Then, all the fittings shown in this paper neglected the differences existing between the commanded and the real gate opening and used the generated signal u(t) as the input to the system, i.e., the exact PRBS. However, the effects of the quantization of the water level measures are noticeable. Then this phenomenon has been included in the models to be identified. Index (19) has been used in order to assess the quality of the obtained models and the matching of their responses to the recorded experimental data.

As a conclusion of this research, two nonlinear fractional order models are proposed for our canal: one that reproduces the free flow regimes, which is given by expression (13), and other that reproduces the submerged flow regimes, which is given by expression (15). These models apply in separate working regions defined by conditions (8) and (10). The parameters of the free flow model are given in Table 7 (nominal model entry) and the parameters of the submerged flow model in Table 4 (nominal model entry). We have tried more complicate models than the ones given by (12)–(15) in order to capture the small oscillations of the responses. Linear and nonlinear models, and integer and fractional order models were tested that completed the already proposed models with sets of lowly damped complex poles and zeros. These models did not show any significant improvement in reproducing these oscillations. For this reason, they have not been presented in this paper.

Finally, we mention that our next works will be to fit hybrid models to the dynamics around a gate opening of 30 mm, in which the two flow regimes appear and to assess if all the obtained results, or part of them, could be applied to the pools of a real irrigation canal. Moreover, implementation issues will be also addressed, see e.g., [39].

## Figures and Tables

**Table 1:** LIOM models identified with the data of Figure 5.

Model	Data	$K_{i}$	$T_{i}$	$L_{i}$	Nrmse
LIOM	op1	0.6806	1.7091	4.9	70.95%
	op2	0.4546	0.2329		64.50%
	op3	0.5576	0.407		65.92%
	op4	0.589	0.7515		76.18%
Nominal		0.5546	0.6829	4.9	op1: 64.63%
					op2: 58%
					op3: 59.14%
					op4: 69.28%
Uncertainties(%)		16.6%	43.19%	-	

**Table 2:** LFOM models identified with the data of Figure 5.

Model	Data	$K_{f}$	$T_{f}$	$\lambda_{f}$	$L_{f}$	Nrmse
LFOM	op1	1.1051	2.057	0.54	4.9	81.21%
	op2	0.8103	1.344	0.35		70.83%
	op3	1.1319	1.795	0.35		75.09%
	op4	0.776	0.856	0.46		80.39%
Nominal		0.9060	1.25	0.41	4.9	op1: 74.63%
						op2: 63.97%
						op3: 64.59%
						op4: 78.62%
Uncertainties(%)		15.72%	29.19%	17.59%	-	

**Table 3:** Parameters of NLIOM-*S* models fitted to op1 and op2 data, and the optimized values of γ.

Model	Data	$K_{i}$	$T_{i}$	$\gamma_{i}$	$L_{i}$	Nrmse
NLIOM-*S*	op1	1.668	2.656	0.47	4.9	81.21%
	op2	1.616	2.084	0.45	4.9	75.09%
Nominal		1.666	2.404	0.45	4.9	op1: 77.32%
						op2: 70.13%
Uncertainties(%)		1.56%	10.77%	2.13%	-	

**Table 4:** Parameters of NLFOM-*S* models fitted to op1 and op2 data and the optimizsed values of γ.

Model	Data	$K_{f}$	$T_{f}$	$\lambda_{f}$	$\gamma_{f}$	$L_{f}$	Nrmse
NLFOM-*S*	op1	1.564	2.313	0.66	0.27	4.9	84.34%
	op2	1.785	1.819	0.48	0.33	4.9	81%
Nominal		1.666	2.404	0.63	0.45	4.9	op1: 82.97%
							op2: 78.36%
Uncertainties(%)		6.19%	10.68%	13.64%	9.09%	-	

**Table 5:** Validation of the obtained models to data obtained in the operating points defined by gate openings [13;23] mm.

Model	${\mathrm{op}}_{1}^{*}$	${\mathrm{op}}_{2}^{*}$
LIOM	64.72%	54.1%
LFOM	73.32%	60.1%
NLIOM-*S*	70.53%	67.05%
NLFOM-*S*	78.81%	78.38%

**Table 6:** Parameters of NLIOM-*F* models fitted to op3 and op4 data, and the optimized values of γ.

Model	Data	$K_{i}$	$T_{i}$	$\gamma_{i}$	$L_{i}$	Nrmse
NLIOM-*F*	op3	1.2295	0.1405	6.15	4.9	71.95%
	op4	0.7532	0.6256	6.85	4.9	78.12%
Nominal		0.7932	0.45	6.15	4.9	op3: 69.64%
						op4: 77.29%
Uncertainties(%)		19.37%	38.77%	5.11%	-	

**Table 7:** Parameters of NLFOM-*F* models fitted to op3 and op4 data, and the optimized values of γ.

Model	Data	$K_{f}$	$T_{f}$	$\lambda_{f}$	$\gamma_{f}$	$L_{f}$	Nrmse
NLIOM-*F*	op3	1.6191	0.6341	0.3	5.8	4.9	75.54%
	op4	0.7798	0.7658	0.57	3.25	4.9	80.4%
Nominal		1.074	0.6805	0.57	5.65	4.9	op3: 75.04%
							op4: 80.33%
Uncertainties(%)		25.92%	8.6%	23.68%	21.98%	-	

**Table 8:** Validation of the obtained models to data obtained in the operating points defined by gate openings [43;45] mm.

Model	${\mathrm{op}}_{3}^{*}$	${\mathrm{op}}_{4}^{*}$
LIOM	63.49%	70.21%
LFOM	65.45%	74.19%
NLIOM-*F*	68.32%	71.15%
NLFOM-*F*	70.8%	76%

## Abbreviations

The following abbreviations are used in this manuscript:

MIMO	Multiple input multiple output
PRBS	Pseudo-random binary signal
LIOM	Linear integer order models
LFOM	Linear fractional order models
NLIOM	Non-linear integer order models
NLFOM	Non-linear fractional order models
ISE	Integral square error
Nrmse	Normalized root mean-squared error
IAE	Integral absolute error
$x_{up}$	Upstream gate level
$y_{dwe}$	Downstream end water level

## Appendix A. Fractional-Order Approximation

The fractional-order integrators and differentiators are implemented based on the Grünwald–Letnikov approximation defined by the following equation:

(A1) $${}_{a}D_{t}^{\alpha }f\left(t\right)=\lim_{h\to 0}\frac{1}{h^{\alpha }}\sum_{j=0}^{\left[\frac{\left(t-a\right)}{h}\right]}{\left(-1\right)}^{j}\left(\begin{array}{c} \alpha \\ j \end{array}\right)f\left(t-jh\right)$$

where [] signifies the integer part, and the combinatorial function has been generalized in the following sense:

(A2) αj=α(α−1)…(α−j+1)j!

where *h* is the step size, D is the differential-integral operator, and α is the fractional-order. Note that the fractional order integrator is implemented using the previous expression with a negative value of α. This discrete operator may be approximated by finite-impulse response(FIR) discrete filters. In this case, expression (A1) is truncated to a fixed number of *N* term of this sum where (N=500). This can be made since limj→∞αj=0, and it is called the short memory approximation. An example of a hardware implementation for solving fractional-order models applying the before mentioned Grünwald–Letnikov approach is detailed in [40]. In this work, the short-memory principle is used, in which the memory length is designed using specialized random access-memory and read-only memory blocks.
